# Perioperative gum-chewing training prevents a decrease in tongue pressure after esophagectomy in thoracic esophageal cancer patients: a nonrandomized trial

**DOI:** 10.1038/s41598-024-74090-4

**Published:** 2024-10-12

**Authors:** Reiko Yamanaka-Kohno, Yasuhiro Shirakawa, Aya Yokoi, Naoaki Maeda, Shunsuke Tanabe, Kazuhiro Noma, Kazuyoshi Shimizu, Toshiharu Mituhashi, Yoshihide Nakamura, Souto Nanba, Yurika Uchida, Takayuki Maruyama, Manabu Morita, Daisuke Ekuni

**Affiliations:** 1 Department of Preventive Dentistry, Division of Dentistry, Okayama University Hospital, 2-5-1 Shikata-cho, Kita-ku, Okayama-shi, Okayama 700-8558 Japan; 2 Department of Gastroenterological Surgery, Okayama University Graduate School of Medicine, Dentistry and Pharmaceutical Science, 2-5-1, Shikata-cho, Kita-ku, Okayama-shi, Okayama 700-8558 Japan; 3 Department of Surgery, Hiroshima City Hiroshima Citizens Hospital, 7-33, Motomachi, Naka-ku, Hiroshima 730-8518 Japan; 4 Department of Preventive Dentistry, Faculty of Medicine, Dentistry and Pharmaceutical Sciences, Okayama University, 2-5-1, Shikata-cho, Kita-ku, Okayama-shi, Okayama 700-8558 Japan; 5 Department of Anesthesiology and Resuscitology, Okayama University Hospital, 2-5-1, Shikata-cho, Kita-ku, Okayama-shi, Okayama 700-8558 Japan; 6 Center for Innovative Clinical Medicine, Okayama University Hospital, 2-5-1, Shikata-cho, Kita-ku, Okayama-shi, Okayama 700-8558 Japan; 7 Department of Oral and Maxillofacial Radiology, Okayama University Graduate School of Medicine, Dentistry and Pharmaceutical Science, 2-5-1, Shikata-cho, Kita-ku, Okayama-shi, Okayama 700-8558 Japan; 8 Dental Clinic, Kurashiki Medical Check-Up Center, 282, Bakurou-cho, Kurashiki-shi, Okayama 710-0824 Japan; 9 Department of Oral Health Sciences, Takarazuka University of Medical and Health Care, 1-1, Midorigaoka, Hanayashiki, Takarazuka-shi, Hyogo 666-0162 Japan

**Keywords:** Esophageal cancer, Esophagectomy, Gum-chewing training, Tongue pressure, Historical control, Propensity score matching analysis, Epidemiology, Oesophageal cancer

## Abstract

Tongue pressure (TP) decreases significantly after esophagectomy in esophageal cancer patients (ECPs). Meanwhile, 2 weeks of gum-chewing training (GCT) significantly increased TP in healthy university students. We examined whether perioperative GCT would decrease the proportion of patients exhibiting a decline in TP at 2 weeks postoperatively, and prevent postoperative complications, in thoracic ECPs (TECPs). This was a single-center interventional study, and nonrandomized study with a historical control group (HCG). TECPs who underwent first-stage radical esophagectomy were recruited. Thirty-two patients of 40 in the gum-chewing group (GCG) were completed perioperative GCT in 3 times daily. Propensity score matching was performed with covariates related to TP including preoperative age, sex, body mass index, and the repetitive saliva swallowing test result, and yielded a matched cohort of 25 case pairs. Eleven GCG patients [44.0%] exhibited significantly lower TP at 2 weeks postoperatively than before esophagectomy was significantly fewer than that of 19 patients [76.0%] in the HCG. The median number of fever days (≥ 38.0 °C) in the 2 weeks after esophagectomy in the GCG was significantly fewer than those in the HCG. Perioperative GCT may prevent postoperative TP decline and postoperative dysphagia-related complications after esophagectomy.

## Introduction

Esophageal cancer is the eighth most common type of cancer and the sixth leading cause of cancer death worldwide^1^. Esophagectomy, which is one of the main treatments, is highly invasive^2^ and is associated with a high risk of postoperative complications including pneumonia, aspiration, recurrent laryngeal nerve palsy, and anastomotic leakage. Skeletal muscle loss is apparent in the acute postoperative phase, worsening the long-term prognosis after esophageal squamous cell carcinoma (SCC) surgery^3^. Although minimally invasive esophagectomy is becoming more popular, efforts are required to improve the prognosis after esophagectomy.

We previously reported that tongue pressure (TP) significantly decreased at 2 weeks postoperatively in esophageal cancer patients (ECPs), and was associated with a longer intensive care unit (ICU) stay and more dysphagia and pneumonia^4^. TP can be easily measured, and a low TP indicates oral hypofunction. Decreased TP is a symptom of dysphagia^5^. Moreover, decreased TP is the only modifiable predictor of aspiration after esophagectomy; rehabilitation that prevents a decrease in TP might reduce the risk of aspiration^6^. It is important to prevent a decrease in TP in perioperative ECPs.

Gum-chewing training (GCT) is a simple way to improve oral function. Two weeks of GCT markedly increased TP in healthy university students^7^. Gum chewing (GC) increased swallowing frequency and reduced swallowing latency in patients with stage 2–4 Parkinson disease who did not exhibit significant prandial dysphagia^8^. In addition, GCT has been found to enhance recovery after surgery. For example, postoperative GC improved gastrointestinal functional recovery in patients who had undergone colorectal surgery and caesarean section^9^. GC serves as an inexpensive, well-tolerated, safe, and effective means of ameliorating ileus after colorectal surgery^10^. GCT may improve various oral functions, including swallowing, and enhance the recovery of gastrointestinal function. However, it remains unknown whether perioperative GCT effectively prevents the TP decrease that develops after esophagectomy.

Against this background, we hypothesized that GCT would prevent the decline in TP after esophagectomy. The purpose of study was to examine whether perioperative GCT would prevent the decline in TP at 2 weeks after esophagectomy and improve the prognosis.

## Materials and methods

### Human rights statement

This interventional study, which compared between gum-chewing group (GCG) and historical control group (HCG), was approved by the Ethics Committee of the Okayama University Graduate School of Medicine, Dentistry and Pharmaceutical Sciences and Okayama University Hospital (no. R 2009-007, 28/07/2020). All procedures were performed in accordance with the ethical standards of all relevant committees on human experimentation (both institutional and national) and the Helsinki Declaration of 1964 and later versions thereof. Written informed consent was obtained from each participant prior to study initiation.

### Study design

This was a nonrandomized controlled study with an HCG. The Patient (P), Intervention (I), Comparison (C), Outcome (O) model was as follows. P: primary thoracic esophageal cancer patients (TECPs) scheduled for one-stage radical esophagectomy; I: perioperative GCT (GCG); C: no perioperative GCT (HCG); and O: the proportion of patients exhibiting a decline in TP at 2 weeks postoperatively. This study was registered (No. UMIN000038361, first registration 01/04/2020) in the University Hospital Medical Information Network, which records academic activities in Japan.

### Sample size calculation

In our previous study, TP decreased significantly from baseline (35.6 ± 7.3 kPa) to 2 weeks postoperatively (34.2 ± 7.3 kPa) (*p* = 0.011, the paired t test), and TP in 37 patients (62.7%) of 59 patients decreased^4^. Among 59 patients, there was 1 patient whose TP was same at baseline and 2 weeks postoperatively (data not shown). Therefore, TP in 38 (64.5%) of 59 patients decreased or unchanged 2 weeks postoperatively^4^. Based on previous our study, we expected that TP in only 40% of the patients decreased postoperatively in GCG^4^. And then, we would have liked to have at most 65% of the proportion of the patients whose TP decreased postoperatively. Thus, the expected and threshold proportions were 40% and 65%, respectively. To ensure an alpha (α) level of 0.05 and a beta (β) level of 0.2, a sample size of 24 was required for a single-arm study. As we employed propensity-score matching with historical controls, it was assumed that 40% of the participants might either remain unmatched or withdraw from the study. This increased the required sample size to 40, which was deemed achievable.

### Study participants

The inclusion criteria were primary TECPs aged 20–79 years and scheduled for first-stage radical esophagectomy at Okayama University Hospital, the ability to understand the intervention, and the provision of voluntary written informed consent after a thorough explanation of the study. The exclusion criteria were an inability to chew gum, patients considered inappropriate, for example, poor adherence, by the principal investigator or the co-investigators, and patients whose physical condition declined after esophagectomy, patients who underwent postoperative swallowing rehabilitation supervised by speech therapists, and patients with both cerebrovascular disorders and dysphagia.

### GCT

All included patients underwent perioperative GCT for about 5 min using two pieces of POs-Ca gum (Ezaki Glico Co., Ltd. Osaka, Japan) three times daily as follows. First, the gum was chewed 10 times on the left and right sides, alternately (3 min)^7^. The tongue was then stretched; the gum was pushed on to the palate five times and saliva was swallowed once. Finally, with the gum stuck to the palate, the lingual frenum was stretched five times^11^. All patients recorded whether GCT was performed in GCT diaries. Training commenced at the preoperative outpatient consultation, or after neoadjuvant chemotherapy in the case of hospitalized patients, was paused on the day of esophagectomy, and restarted no earlier than 2 days later only after the esophageal surgeon and the anesthesiologist confirmed that it was safe to do so. The duration of preoperative GCT depended on the day on which the patient commenced GCT, and that of postoperative GCT depended on the day on which GCT was restarted (maximum 13 days).

### TP evaluation and repetitive saliva swallowing test (RSST)

The TP and RSST score were measured on the day before esophagectomy and at 2 weeks postoperatively. The TP was measured as the tongue pressed the balloon of the TP instrument (JMS Co. Ltd., Hiroshima, Japan)^12^. The mean of three measurements was recorded. The change in TP was calculated by subtracting the TP on the day before esophagectomy from that at 2 weeks after esophagectomy. In the RSST, patients were instructed to swallow as many times as they could within 30 s, and the number of swallows was counted. Dysphagia was suspected if the RSST score was < 3^13^. In this study, the RSST score was treated as a continuous variable.

### General patient status

Patients’ preoperative characteristics and postoperative clinical findings were recorded on electronic medical charts and are summarized in Tables 1 and 2. Pneumonia, aspiration, and anastomotic leakage were considered present ( +) when the Clavien–Dindo (CD) grade was ≥ 2. Postoperative recurrent laryngeal nerve palsy was considered present ( +) if the CD grade was ≥ 1 (clinical observation or diagnostic evaluation only; intervention not indicated)^6,14–16^. Vocal cord paralysis was confirmed by postoperative fiberoptic examination or fluoroscopy.

**Table 1:** Preoperative characteristics before and after propensity score matching.

Characteristics	Entire cohort		SMD
	GCG (n = 32)	HCG (n = 44)	
Age, median [year (IQR)]	70.0 (60.3–73.0)	64.0 (61.0–68.8)	0.21
Sex, Males (%)/Females (%)	27 (84.4)/5 (15.6)	31 (70.5)/13 (29.5)	0.16
BMI, median [kg/m^2^ (IQR)]	23.1 (20.6–25.5)	21.6 (19.9–23.2)	0.23
Preoperative RSST score	5.0 (4.0–6.8)	4.0 (3.3–5.0)	0.43
Preoperative tongue pressure [kpa (IQR)]	34.9 (30.5–40.3)	35.4 (29.9–41.3)	0.03
Neoadjuvant chemotherapy ( +)	23 (71.9)	28 (63.6)	0.09
Clinical stage (8th UICC) 0, I, II (%)/III, IV (%)	18 (56.3)/14 (43.8)	31 (70.5)/13 (29.5)	0.15
Histological diagnosis			0.28
SCC (%)/adenocarcinoma (%)/others (%)	26 (81.3)/4 (12.5)/ 2 (6.3)	43 (97.7)/0 (0.0)/ 1 (2.3)	
Smoking status			0.24
Never (%)/Past (%)/Current (%)	3 (9.4)/26 (81.3)/3(9.4)	6 (13.6)/38 (86.4)/0 (0.0)	
Quantity and frequency of drinking (/week)			0.55
Never (%)/Light (%)/Moderate (%)/Heavy (%)	8 (25.0)/4 (12.5)/12 (37.5)/8 (25.0)	5 (11.4)/1 (2.3)/38 (86.4)/0 (0.0)	
Preoperative WBC count [10^3^/µL (IQR)]	5.25 (4.45–6.49)	4.86 (4.08–5.47)	0.20
Preoperative CRP concentration [mg/dL (IQR)]	0.12 (0.05–0.19)	0.12 (0.05–0.21)	− 0.01
Preoperative albumin concentration [g/dL (IQR)]	3.9 (3.6–4.0)	3.9 (3.6–4.2)	− 0.10

Characteristics	Propensity score-matched cohort		SMD
	GCG (n = 25)	HCG (n = 25)	
Age, median [year (IQR)]	70.0 (61.5–73.0)	64.0 (60.5–69.5)	0.19
Sex, Males (%)/Females (%)	21 (84.0)/4 (16.0)	20 (80.0)/5 (20.0)	0.05
BMI, median [kg/m^2^ (IQR)]	23.0 (20.6–25.4)	21.5 (19.5–23.4)	0.22
Preoperative RSST score	5.0 (4.0–6.0)	4.0 (4.0–5.0)	0.24
Preoperative tongue pressure [kpa (IQR)]	35.0 (30.7–41.4)	38.2 (30.4–43.8)	−0.05
Neoadjuvant chemotherapy ( +)	19 (76.0)	15 (60.0)	0.18
Clinical stage (8th UICC)			0.31
0, I, II (%)/III, IV (%)	14 (56.0)/11 (44.0)	21 (84.0)/4 (16.0)	
Histological diagnosis			0.33
SCC (%)/adenocarcinoma (%)/others (%)	20 (80.0)/3 (12.0)/2(8.0)	25 (100)/0 (0.0) /0 (0.0)	
Smoking status			0.08
Never (%)/Past (%)/Current (%)	2 (8.0)/20 (80.0)/3 (12.0)	3 (12.0)/22 (88.0)/0 (0.0)	
Quantity and frequency of drinking (/week)			0.13
Never (%)/Light (%)/Moderate (%)/Heavy (%)	7 (28.0)/3 (12.0)/11 (44.0)/4(16.0)	4 (16.0)/1 (4.0)/20 (80.0)/0 (0.0)	
Preoperative WBC count [10^3^/µL (IQR)]	5.64 (4.52–6.69)	4.87 (4.18–5.32)	0.24
Preoperative CRP concentration [mg/dL (IQR)]	0.12 (0.05–0.19)	0.12 (0.04–0.29)	0.02
Preoperative albumin concentration [g/dL (IQR)]	3.9 (3.7–4.0)	4.0 (3.7–4.2)	−0.12

*GCG* gum-chewing group*, HCG* historical control group, *IQR* interquartile range, *BMI* body mass index, *RSST* repetitive saliva swallowing test, *UICC Union for International Cancer Control*, *SCC* squamous cell carcinoma, *WBC* white blood cell, *CRP* C-reactive protein, *SMD* standardized mean difference.

**Table 2:** Clinical findings after esophagectomy.

Characteristics	GCG (n = 25)	HCG (n = 25)	*p* Value
Operative approach, thoracotomy (%)/thoracoscopy (%)	3 (12.0)/22 (88.0)	5 (20.0)/20(80.0)	0.351^b^
Lymphadenectomy, two- field dissection (%)/three-field dissection (%)	0 (0.0)/25 (100.0)	8 (32.0)/ 17 (68.0)	0.002^b^
Operative time [min (IQR)]	650.0 (572.0–697.0)	549.0 (504.5–579.0)	0.001^a^
Operative bleeding [mL (IQR)]	130.0 (80.0–222.5)	150.0 (95.0–325.0)	0.327^a^
Intubation days [days (IQR)]	1.0 (0.0–1.0)	1.0 (1.0–1.0)	0.005^a^
Postoperative fasting days [days (IQR)]	11.0 (9.0–13.5)	9.0 (8.0–12.0)	0.036^a^
Postoperative pneumonia ( +)	5 (20.0)	5 (20.0)	0.637^b^
Postoperative aspiration ( +)	0 (0.0)	3 (12.0)	0.117^b^
Postoperative recurrent laryngeal nerve palsy ( +)	4 (16.0)	2 (8.0)	0.334^b^
Postoperative anastomotic leakage ( +)	1 (4.0)	0 (0.0)	0.500^b^
ICU stays [day (IQR)]	4.0 (4.0–4.0)	6.0 (5.0–6.0)	0.000^a^
Postoperative hospital stays [day (IQR)]	23.0 (20.0–27.5)	19.0 (16.0–21.5)	0.005^a^
Postoperative WBC count [10^3^/µL (IQR)]	6.67 (5.68–8.20)	6.60 (5.81–8.00)	0.923^a^
Postoperative CRP concentration [mg/dL (IQR)]	0.44 (0.30–1.13)	0.74 (0.315–2.485)	0.277^a^
Postoperative albumin concentration [g/dL (IQR)]	3.2 (3.0–3.4)	3.2 (3.1–3.4)	0.952^a^
BMI at 2 weeks after esophagectomy, median [kg/m^2^ (IQR)]	22.4 (20.1–24.0)	20.9 (18.8–22.1)	0.086^a^
RSST score at 2 weeks after esophagectomy (IQR)	5.0 (3.5–6.0)	4.0 (2.5–4.0)	0.004^a^
Tongue pressure [kPa (IQR)]	37.6 (31.7–41.7)	33.0 (28.8–38.7)	0.157^a^

*GCG* gum-chewing group, *HCG* historical control group, *ICU* intensive care unit, *IQR* interquartile range, *BMI* body mass index, *RSST* repetitive saliva swallowing test,

^a^Mann–Whitney *U* test, ^b^Fisher’s exact test.

### Covariates

We identified potential confounders that were plausibly associated with TP based on clinical knowledge and previous studies^17–19^. After consideration, preoperative age, sex, body mass index (BMI), and RSST score were selected as potential confounders.

### Statistical analyses

Data are presented as frequencies with proportions or as medians with interquartile range (IQR; 25% and 75% quartiles), as appropriate.

The research question was whether the outcomes of the gum-chewing group were affected by GCT. Adjusted TP differences were obtained using propensity-score matching. The GCT propensity score was estimated using a logistic regression model including potential confounders. GCG patients (n = 32) were matched to HCG patients (n = 44) using a 1:1 nearest-neighbor matching algorithm with replacement with a caliper of 0.35 for the standard deviation (SD) of the propensity score (logit scale). The recommended caliper size is 0.2 SD^20^, but in this study 0.35 SD was used because this was an exploratory analysis. The “between-group covariate balance” was assessed before and after matching; we considered an absolute standardized mean difference (SMD) < 0.25 to be indicative of balance^21^. We used robust standard errors to accommodate the matching.

The Mann–Whitney *U* test, chi-squared test, and Fisher’s exact test were used as appropriate to compare the two groups. A *p*-value < 0.05 was considered significant. All statistical analyses were performed with IBM SPSS Statistics for Windows (version 26.0; IBM Japan, Tokyo, Japan).

## Results

Forty TECPs were enrolled in this study between 29 March 2021 and 19 May 2022. All HCG subjects were enrolled between January 2016 and December 2017^4^.

**Fig. 1:**
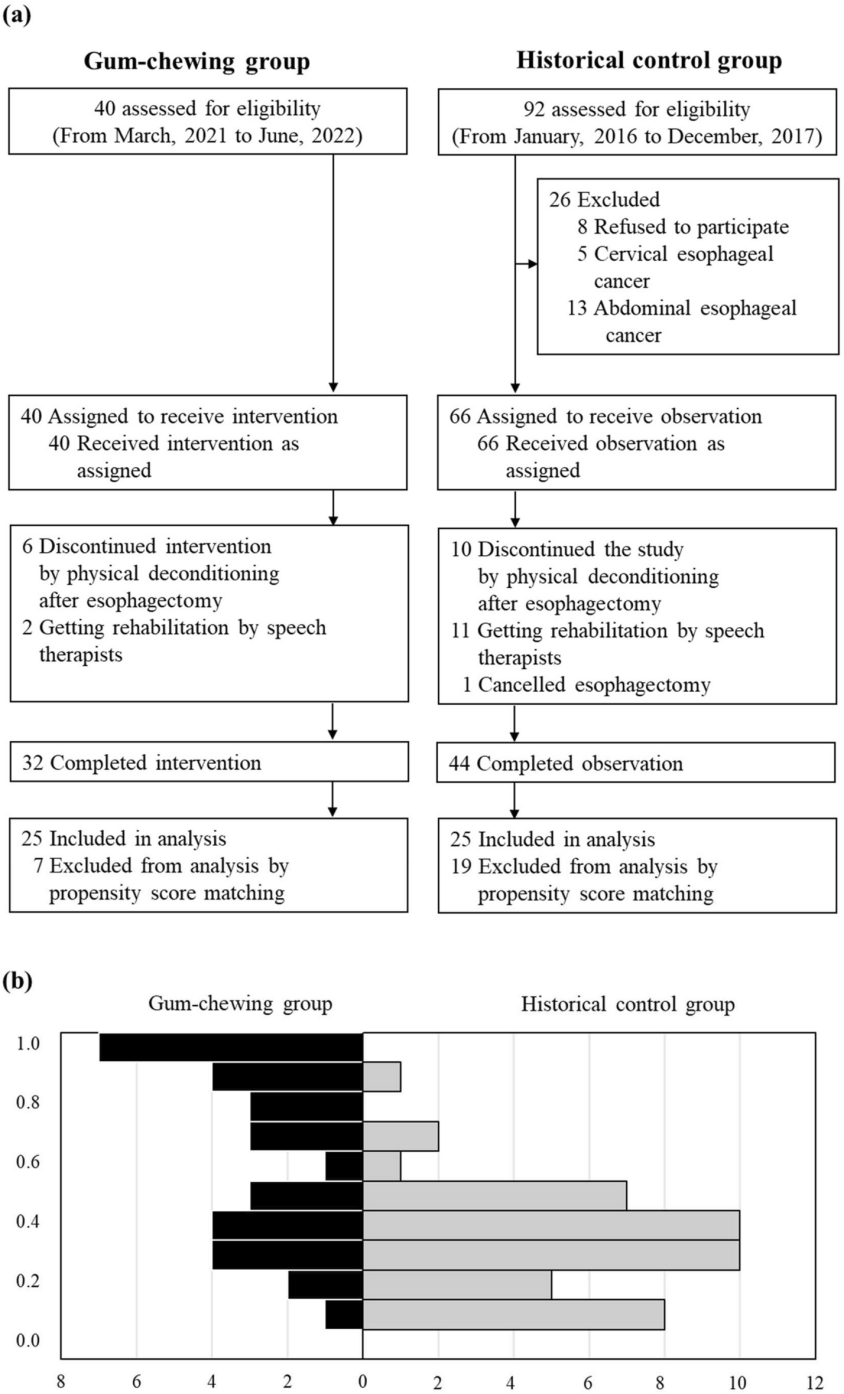
Patient flow chart and Distribution of propensity scores between groups. (**a**) Patient flow chart. (**b**) Distribution of propensity scores between groups.

Figure 1A shows that 32 [27 males and 5 females, aged 41–78 years; mean age = 66.4 years, median age = 70.0 years] of 40 patients completed the study. The follow-up rate was 80.0% (32/40) in the GCG and 66.7% (44/66) in the HCG. The GCT duration before and after esophagectomy was 16.0 (IQR 10.5, 22.0) and 13.0 (IQR 13.0, 13.0) days, respectively. The GCT rate before and after esophagectomy was 92.2% (IQR 86.5, 100.0%) and 79.5% (IQR 55.9, 92.8%), respectively. There was one adverse event; one patient’s dental restoration came free during GCT. The restoration was reglued, and the patient continued GCT.

Propensity-score matching yielded a matched cohort of 25 case pairs (Table 1). Matched subjects were similar; the SMDs of all potential confounders were < 0.25. The c-statistic indicating that propensity scoring predicted the need for intervention was 0.694, indicating relatively low discrimination. The propensity-score distributions differed between the groups; the common support range was 0.1–0.7 (Fig. 1B).

**Fig. 2:**
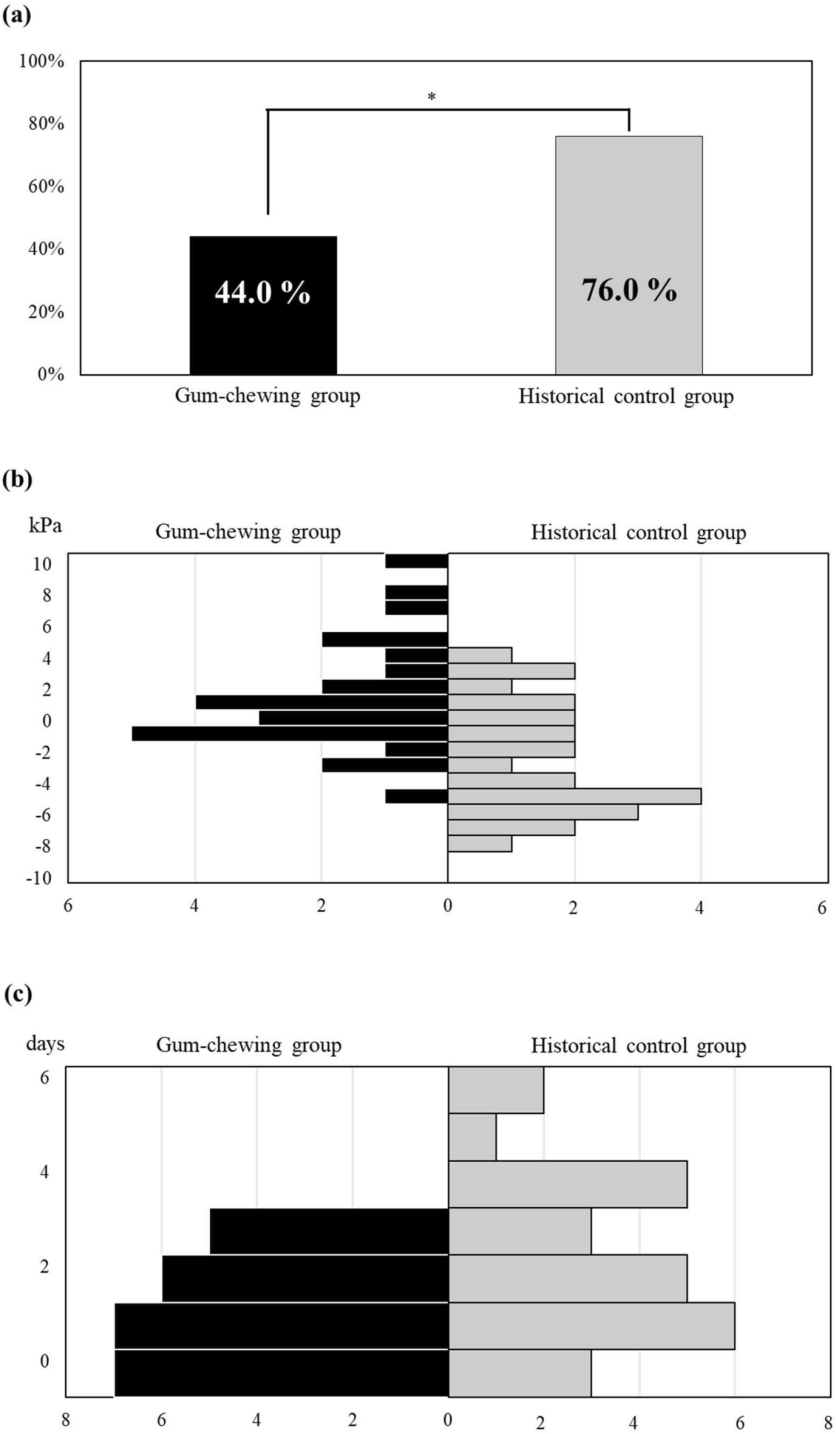
Proportion of patients and histograms for gum-chewing group (GCG) and historical control group (HCG). (**a**) Proportion of patients with decreased tongue pressure (TP) at 2 weeks postoperatively (*p = 0.020, Fisher’s exact test), (**b**) Value obtained by subtracting the TP on the day before esophagectomy from that at 2 weeks postoperatively (p = 0.001, Mann–Whitney *U* test), *GCG* Median value, 0.77; interquartile range, − 1.6–3.2, *HCG* Median value, − 3.1; interquartile range, − 6.1 to − 0.3, (**c**) Number of fever (≥ 38.0 °C) days during 2 weeks postoperatively (*p* = 0.025, Mann–Whitney *U* test), *GCG* Median value, 1; interquartile range, 0–2, *HCG* Median value, 2; interquartile range, 1–4.

In the GCG, the proportion of patients exhibiting a decreased TP at 2 weeks postoperatively was 44.0%, significantly lower than that in the HCG (76.0%; Fig. 2A). The change in TP was significantly greater in the GCG than in the HCG (*p* = 0.03) (Fig. 2B). The median number of postoperative fever days was significantly lower in the GCG than in the HCG (Fig. 2C).

Table 2 showed that the clinical findings after esophagectomy in GCG and HCG. The proportion of patients who underwent three-field dissection lymphadenectomy, the operative time, the postoperative fasting and hospital days, and the RSST score at 2 weeks postoperatively were all significantly higher in the GCG than in the HCG. The number of intubation days and the length of ICU stay were significantly longer in the HCG than in the GCG.

## Discussion

The proportion of patients exhibiting decreased postoperative TP was significantly lower in the GCG than in the HCG group, and TP increased after esophagectomy in more than half of patients in the GCG. Moreover, the median number of postoperative fever days was significantly lower in the GCG than in the HCG. To the best of our knowledge, this is the first study to show that perioperative GCT for TECPs prevented postoperative decline in TP, reduced the number of postoperative fever days, and maintained or improved swallowing capacity. Perioperative GCT is considered safe, because GCT did not increase the incidence of postoperative complications in the GCG.

This study supports the results of a previous study that 2 weeks of GCT significantly increased the TP of university students^7^. The duration of postoperative GCT was about 2 weeks. GCT training was performed even during the postoperative fasting period and thus improved TP postoperatively. As the outcomes of university students and perioperative TECPs were similar, the observed effect of GCT on TP was considered to have external validity.

The median number of postoperative fever days was significantly lower in the GCG than in the HCG, possibly because none of the GCG patients developed postoperative aspiration. In fact, the median postoperative RSST score was significantly higher in the GCG than in the HCG. Swallowing function was improved by perioperative GCT, which may have prevented postoperative aspiration. Post-esophagectomy pneumonia is at least partly attributable to swallowing dysfunction and silent tracheobronchial aspiration; both develop at relatively high rates among esophagectomy patients in the early postoperative period^22^. As well as pneumonia, fever without active infiltration on chest X-rays and computed tomography may reflect aspiration^23^. Postoperative aspiration in ECPs is positively associated with older age, a higher likelihood of recurrent postoperative laryngeal palsy, a lower postoperative TP, and a greater decrease in postoperative TP^6^. The incidence of recurrent laryngeal palsy tended to be higher in the GCG than in the HCG. Nevertheless, postoperative aspiration did not occur in the GCG. GCT may enhance swallowing function, including TP; this may overcome recurrent laryngeal palsy. Thus, perioperative GCT may reduce the risk of aspiration and postoperative fever.

It remains unclear whether perioperative GCT prevents postoperative aspiration and pneumonia in TECPs, because the incidence rates there of did not differ significantly between GCG and HCG. If the primary outcomes of an interventional study were postoperative aspiration and pneumonia rates, the effect of GCT would become clearer. However, a larger-scale study is required because the incidence rates are low. In this study, the primary outcome was prevention of the postoperative decline in TP. The sample size was small, and we used propensity-score matching to reduce confounding. The propensity-score-matching caliper was widened to 0.35 from the usual 0.2, yielding SMDs of 0.25. However, the SMDs of age, sex, BMI, and the preoperative RSST score, all of which affect TP, were all < 0.25, and that of the preoperative TP was < 0.1. Although the propensity-score matching was thus not completely effective, this exploratory study nonetheless yielded valuable results.

Contrary to our expectations, the median number of postoperative fasting days and the duration of hospital stay were significantly longer in the GCG than in the HCG. These results are explained by the different recruiting periods for the GCG and HCG subjects. All GCG patients were enrolled in 2021 and 2022 during the global coronavirus disease 2019 (COVID-19) pandemic. During that time in Japan, the proportion of ECPs with stage 0–II disease decreased and that of patients with stage III or IV disease increased, although statistical significance was not attained^24^. The proportion of patients with clinical stage III–IV disease was considerably higher in the GCG than in the HCG. Moreover, all GCG patients underwent lymphadenectomy with three-field dissection, and surgery times were thus significantly longer than those of the HCG. In other words, the GCG patients had more advanced esophageal cancers compared to the HCG patients and underwent more invasive esophagectomies. It is thus reasonable that the GCG patients fasted for longer after esophagectomy and required longer hospital stays than the HCG patients. Nevertheless, perioperative GCT clearly prevented the postoperative decline in TP; this is important, in that the risks of postoperative aspiration and pneumonia, both of which are serious complications, were potentially reduced.

The median length of ICU stay was significantly shorter in the GCG than in the HCG, in line with our previous finding that a greater postoperative decrease in TP was associated with a longer ICU stay^4^. The use of perioperative GCT to improve postoperative TP may have prevented postoperative aspiration and subsequent fever, thus reducing the ICU stay. Recently, to enhance recovery after esophagectomy, we have tried to extubate either on the day of esophagectomy or on the next day. GCT in combination with this change might have contributed to reducing the length of ICU stay. As another reason, it might had better be considered that the brevity of ICU stays in gum chewing group might have affected by COVID-19 pandemic. Because bed in ICU should have been emptied as soon as possible for the very severe COVID-19 patients. From these reasons, both shortening of intubation days and ICU stay might have been affected by the recruiting period of subjects. Moreover, those are undeniable that shortening of intubation days and ICU stay might have been affected on postoperative TP.

Implementing GCT may improve patients’ prognosis without increasing the burden imposed on medical staff. The dropout rate tended to be lower in the GCG than in the HCG. Fewer patients dropped out due to rehabilitation by speech therapists in the GCG than in the HCG (2/40 [5.0%] and 11/66 [16.7%], respectively). The proportion of patients who required speech therapists-aided swallowing training might have been lower in the GCG than in the HCG because GCT maintained swallowing function. Our multidisciplinary team reported that our professional support contributed to the good prognosis after esophagectomy^25–29^. On the other hand, GCT does not require a professional. Thus, perioperative GCT may be very cost-effective in terms of TECPs’ rehabilitation.

Perioperative GCT may improve quality of life (QOL) by preventing sarcopenia after esophagectomy. Sarcopenia is a risk factor for worsening global QOL, reduced physical and role functions, and severe fatigue at 4 weeks after esophageal cancer surgery^30^. Many studies have described the relationship between low TP and sarcopenia. Older adults who were frail or exhibited sarcopenia had lower TPs^31^. Decreased TP is associated with sarcopenia and sarcopenic dysphagia in the elderly^32^. A low TP may increase the risk of malnutrition^33^. Thus, prevention of any postoperative TP decline might improve QOL by enhancing nutrition and preventing sarcopenia. Thus, GCT could be useful for policies that seek to promote healthy aging, as it is safe and effective, even for patients at extremely high risk of aspiration after esophagectomy.

This study had some limitations. First, it was not a randomized controlled study, and some biases and confounding factors may not have been adequately controlled for. For example, the HCG was recruited about 5 years before the GCG. We thus performed propensity-score matching of four covariates: preoperative age, sex, BMI, and the RSST score. However, it remains possible that the balancing was insufficient. Second, there may have been some unmeasured confounders because we adjusted for only four confounders. Third, this study was performed in only one hospital, and all patients were ethnically Japanese. A randomized, global multicenter clinical trial is required.

In conclusion, we found that perioperative GCT safely prevented the postoperative decrease in TP in TECPs and reduced the number of postoperative fever days. Perioperative GCT is a simple and safe oral rehabilitation technique that may prevent dysphagia and sarcopenia and improve the QOL of TECPs.

## Data Availability

The data supporting the results of this study are available from the corresponding author upon reasonable request.
